# Wireless Power Delivery Techniques for Miniature Implantable Bioelectronics

**DOI:** 10.1002/adhm.202100664

**Published:** 2021-06-10

**Authors:** Amanda Singer, Jacob T. Robinson

**Affiliations:** ^1^ Department of Electrical and Computer Engineering Rice University 6100 Main St Houston TX 77005 USA

**Keywords:** bioelectronics, implanted devices, review, wireless power transfer

## Abstract

Progress in implanted bioelectronic technology offers the opportunity to develop more effective tools for personalized electronic medicine. While there are numerous clinical and pre‐clinical applications for these devices, power delivery to these systems can be challenging. Wireless battery‐free devices offer advantages such as a smaller and lighter device footprint and reduced failures and infections by eliminating lead wires. However, with the development of wireless technologies, there are fundamental tradeoffs between five essential factors: power, miniaturization, depth, alignment tolerance, and transmitter distance, while still allowing devices to work within safety limits. These tradeoffs mean that multiple forms of wireless power transfer are necessary for different devices to best meet the needs for a given biological target. Here six different types of wireless power transfer technologies used in bioelectronic implants—inductive coupling, radio frequency, mid‐field, ultrasound, magnetoelectrics, and light—are reviewed in context of the five tradeoffs listed above. This core group of wireless power modalities is then used to suggest possible future bioelectronic technologies and their biological applications.

## Graphical abstract

Six different types of wireless power transfer technologies used in bioelectronic implants—inductive coupling, radio frequency, mid‐field, ultrasound, magnetoelectrics, and light—in the context of five fundamental tradeoffs: power, miniaturization, depth, alignment tolerance, and transmitter distance are reviewed. Then possible future bioelectronic technologies and their biological applications for these wireless power modalities are suggested.

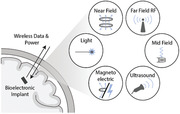

## 1 Introduction

Advances in bioelectronic medicine are continually driven by new developments in targeted electronic devices that stimulate and sense physiological processes in the body. In humans we see examples of these technologies in devices such as pacemakers, brain electrodes, glucose monitors, cochlear implants, and spinal cord stimulators. Recent progress in materials and fabrication resulted in new devices that are softer and more flexible and electrodes that have lower impedance.^[^ ^1^, ^2^, ^3^ ^]^ This variety of tools allows researchers and clinicians to tailor bioelectronics for specific applications in humans and animal models. Similarly, we believe there are similar opportunities for materials advances to improve the way we deliver power to these bioelectronic devices.

**Figure 1:**
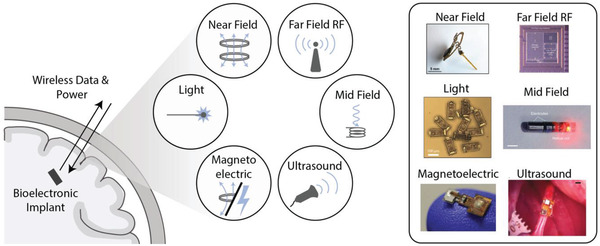
Different power modalities for wireless bioelectronic implants.^[^ ^10^, ^11^, ^12^, ^13^, ^14^, ^15^ ^]^ Adapted with permission.^[^ ^13^ ^]^ Copyright 2017, IEEE;^[^ ^15^ ^]^ Copyright 2017, Springer Nature;^[^ ^16^ ^]^ Copyright 2020, IEEE;^[^ ^11^ ^]^ Copyright 2020, Springer Nature.

Wireless, battery‐free technologies offer a number of advantages for both preclinical testing and clinical applications. Most bioelectronic implants currently used in a clinical setting are battery powered, often with leads extending from an implanted pattern generator (IPG) to the stimulation target. While battery power ensures that these devices are reliably powered, it also adds surgical complexity due to the larger device size and introduces potential issues with lead migration and disconnection over time.^[^ ^4^, ^5^, ^6^ ^]^ It also limits the placement of devices to areas where leads can be placed between the battery and the target tissue, or, in the case of leadless batteries, to surgically accessible areas where the device will fit.^[^ ^7^, ^8^ ^]^ Wireless, battery‐free devices are generally smaller and can be placed in specific, previously inaccessible, locations without lead wires. Furthermore, in preclinical work in animal models, especially rodent models, there is a need to interface with even smaller target areas which are impractical to target with large battery powered devices. In some cases, tethered devices, where an external supply powers an implanted device, are sufficient, however these tethers can interfere with natural behavior and are challenging to use for chronic studies.^[^ ^9^ ^]^ Miniature wireless devices offer advantages of accessing small target areas in ways that allow for unimpeded motion (**Figure** **1**).

**Figure 2:**
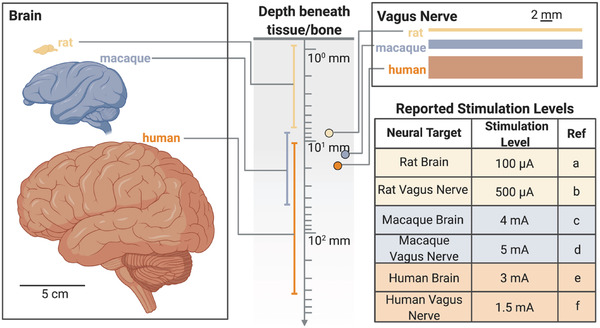
Size and depth differences in anatomical targets lead to a need for a variety of wireless power schemes for implanted bioelectronics. (References a,^[^ ^23^, ^24^ ^]^ b,^[^ ^11^ ^]^ c,^[^ ^19^ ^]^ d,^[^ ^22^ ^]^ e,^[^ ^21^ ^]^ f^[^ ^20^ ^]^)

Technology challenges for wireless power delivery often depend on whether one is working with a small model, large animal, or human, and where in the body the implant is located. For example, the geometry of the nervous system varies dramatically across humans and animal models (**Figure** **2**). A rodent brain is only 1–2 cm long, while a human brain is 10–15 cm.^[^ ^17^ ^]^ Similarly, a rodent vagus nerve is significantly smaller than a human or larger animal model vagus nerve.^[^ ^18^ ^]^ In a simple sense, this means that technology designed for larger animals may not need to be miniaturized to the same degree as a device designed for a mouse; however, the wireless power may need to propagate through a significantly greater distance in bone and tissue. On the other hand, bioelectronics designed for rodent models must be small and lightweight but need not operate at the same depths within the tissue. Furthermore, the electrical current levels required to stimulate various nerves electrically also differ depending on the animal and target application (Figure 2).^[^ ^19–11^ ^]^ This naturally existing intricacy in biological systems adds extra layers of complexity to developing wireless bioelectronic devices. As a result, optimal wireless power transfer (WPT) technologies clearly change depending on the animal model or clinical target.

Safety limits and crossing between different media like air and bone add to the challenges facing wireless data and power transfer to implanted bioelectronic devices.^[^ ^25^ ^]^ Wireless links between a transmitter and receiver are significantly less efficient than a direct wired connection and much of the transmitted power is lost along the path between the implant and transmitter. This can be due to the geometry of the system as well as reflected power due to impedance mismatches at the boundaries of air and different types of tissue. In order to account for this and deliver a suitable amount of power to the implant, systems will increase the amount of power applied by the transmitter. However, in doing this the human body cannot be exposed to excessive amounts of heat or electric field. These safety requirements restrict the applied power levels and thus help to set the maximum power available to the implant. Many types of implants can also require an onboard charge storage capacitor to deliver sufficient charge to an implant which can limit the operating frequency of the device and increase the device footprint.^[^ ^11^, ^26^ ^]^

**Figure 3:**
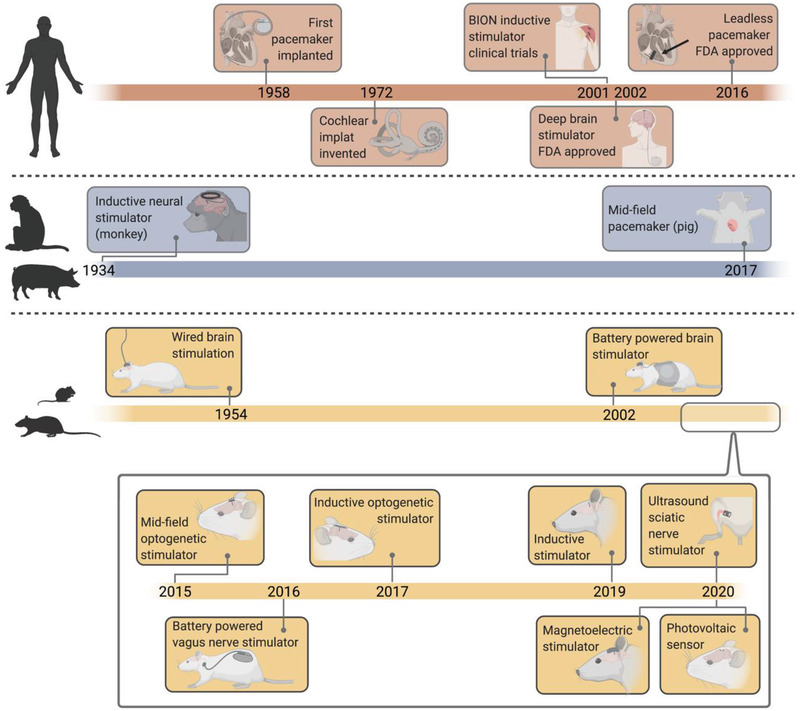
Timeline of bioelectronic power delivery methods in various clinical and preclinical applications shows a variety of recently proposed wireless power delivery solutions, especially in rodent models.^[^ ^12^, ^15^, ^23^, ^24^, ^26^, ^27^, ^28^, ^29^, ^30^, ^31^, ^32^, ^33^, ^34^, ^35^, ^36^ ^]^

To overcome these challenges researchers have developed a number of wireless data and power transfer technologies for implanted bioelectronic devices, each with advantages and disadvantages when compared to alternative approaches (**Figure** **3**). Over time, devices have generally been made smaller and more efficient moving from devices that could only be used in large animals to millimeter‐sized devices compatible with rodents (Figure 3).^[^ ^12^, ^15^, ^23^, ^24^, ^26^, ^27^, ^28^, ^29^, ^30^, ^31^, ^32^, ^33^, ^34^, ^35^, ^36^ ^]^

**Figure 4:**
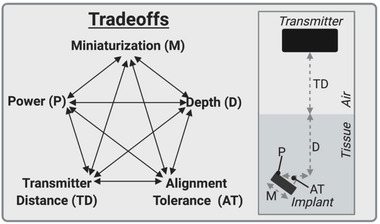
Five fundamental tradeoffs for wireless power delivery.

With the expanded library of WPT technologies we sought to understand the strengths and weaknesses of each approach by comparing them based on five metrics that are typically the major design considerations for bioelectronic implants: miniaturization, depth, alignment tolerance, transmitter distance, and power (**Figure** **4**).

We define miniaturization as the longest length of the wireless power receiver on the implant (which usually correlates with the longest length of the device). Depth refers to the depth in tissue an implant can be, which is often directly related to the safety limits of the power type or the geometry of the device. Alignment tolerance is the sensitivity of the device to a combination of angular and translational misalignment. We estimated this metric by multiplying the approximate angular alignment tolerance by the approximate translational alignment tolerance. We define the angular alignment tolerance as the fraction of angles between 0 and 90 where the device receives sufficient power to function. Similarly, we define the translational alignment tolerance as the fraction of the area of a 10 cm diameter circle within which translational misalignment still results in the device receiving sufficient power to function (up to 5 cm translational displacement in any direction). Transmitter distance is the distance the wireless power can reliably transfer through air, which is especially important in freely moving rodent applications. Power refers to the power the implant can generate. While these factors are all interdependent to a point (i.e., a smaller or deeper implanted device will generate less power), there are also fundamental limitations for each type of wireless power technique which allow us to estimate the design space available for each different modality. Here we focus on near field inductive coupling, far field antenna, mid‐field inductive coupling, ultrasound, magnetoelectric, and photovoltaic techniques since these technologies have been demonstrated in vivo.

**Figure 5:**
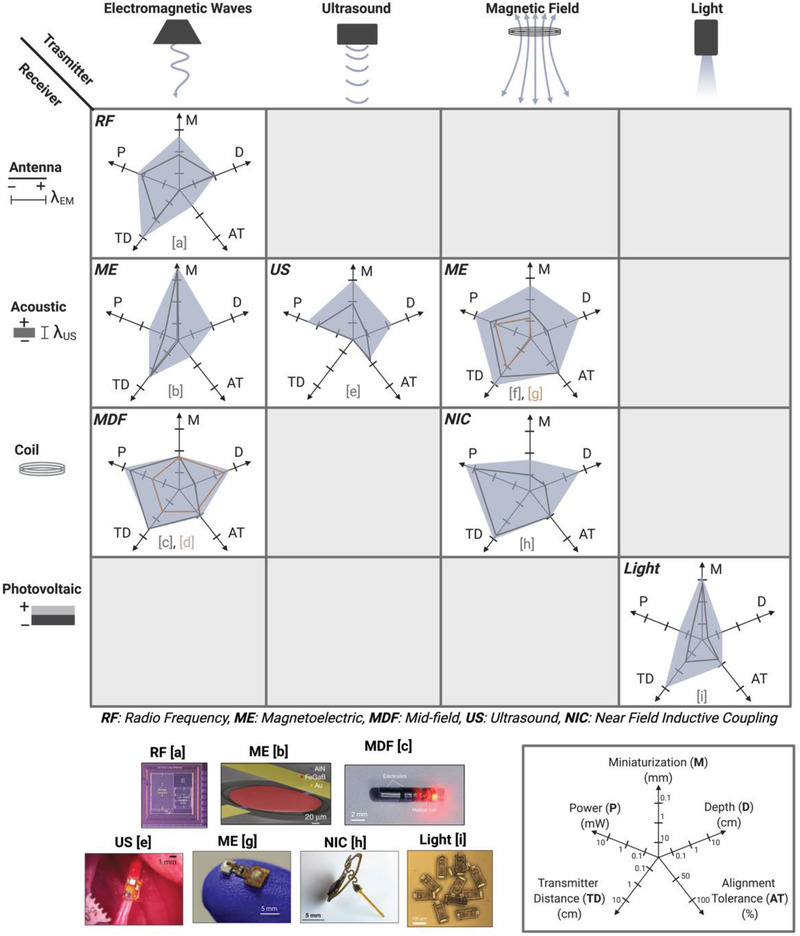
Comparison of wireless power delivery methods based on five metrics: Miniaturization (M), Depth (D), Alignment Tolerance (AT), Transmitter Distance (TD), and Power (P) shows the possible application spaces for each of the six major forms of WPT. (References a,^[^ ^13^ ^]^ b,^[^ ^37^ ^]^ c,^[^ ^15^, ^38^ ^]^ d,^[^ ^29^ ^]^ e,^[^ ^11^ ^]^ f,^[^ ^23^ ^]^ g,^[^ ^39^ ^]^ h,^[^ ^14^ ^]^ i^[^ ^12^ ^]^) Adapted with permission.^[^ ^13^ ^]^ Copyright 2017, IEEE;^[^ ^15^ ^]^ Copyright 2017, Springer Nature;^[^ ^16^ ^]^ Copyright 2020, IEEE;^[^ ^11^ ^]^ Copyright 2020, Springer Nature. Adapted under the terms of the CC BY license.^[^ ^37^ ^]^ Copyright 2017, the Authors. Published by Springer Nature.

To visually compare the main WPT technologies for bioelectronics we categorized the technologies based on the type of transmitter and the type of receiver and compare their performance according to the 5 metrics described above (**Figure** **5**). The four basic transmitter types used to send wireless power into the body are electromagnetic antennas, ultrasound transducers, magnetic coils, and light (usually infrared). These types of transmitters couple power into an implant that contains either an antenna, acoustic material, coil, or photovoltaic component. We then plotted each of the five metrics on a separate axis with our estimation of where the fundamental limits for each factor lies based on the physics of each technique and the limits imposed by biological geometry and safety. It should be noted that the shaded boundary represents our estimated limit for each metric and not the space that any particular device would occupy. In other words, we expect any instantiation of a wireless device to fall within the shaded region. As examples, we plotted the performance of numerous devices reported in the literature on the radar plots showing that they all fall within the shaded regions. While we note that many of these devices are designed for a specific bioelectronic application, we aim to discuss the geometry and delivered power because these WPT methods could be applied to many potential applications.

In the following sections we explore the fundamentals of the six major WPT technologies considered here and discuss other WPT methods before concluding with a look toward the future of miniature wireless bioelectronics.

## 2 Wireless Power Delivery Methods

### 2.1 Near Field Inductive Coupling

**Figure 6:**
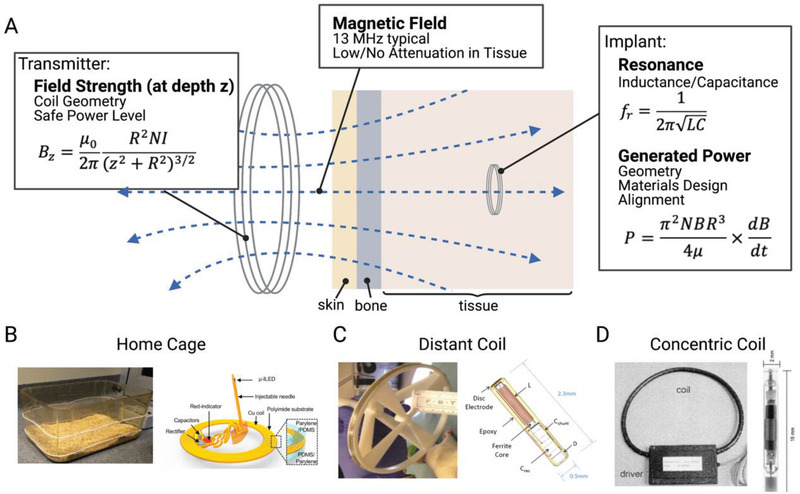
Near field inductive coupling A) uses a transmitting coil to power a smaller implanted coil using alternating magnetic fields. This type of system can be used in B) home cages,^[^ ^28^ ^]^ C) coils from a distance away,^[^ ^27^, ^42^ ^]^ and D) coils placed around a peripheral limb.^[^ ^26^ ^]^ Adapted with permission.^[^ ^29^ ^]^ Copyright 2017, Elsevier;^[^ ^26^ ^]^ Copyright 2001, Elsevier. Adapted under the terms of the CC BY license.^[^ ^42^ ^]^ Copyright 2017, the Authors. Published by Frontiers.

Near field inductive coupling (NIC) is one of the most commonly used methods of wireless power transfer to implanted bioelectronic devices. This method uses an external coil (or in some cases a loop antenna) to generate an alternating magnetic field that is transferred through tissue to a second, smaller, implanted coil (**Figure** **6**A).^[^ ^14^, ^26^, ^28^, ^40^, ^41^, ^42^ ^]^ This method of wireless power transfer is attractive due to the fact that magnetic fields at these frequencies can safely pass through tissue from a small distance away with little or no attenuation.^[^ ^43^ ^]^ While it is often referred to as radio frequency in literature, the magnetic fields here do not radiate as radio frequency electromagnetic waves. We discuss radio frequency electromagnetic waves for far‐field technologies WPT in a later section.

When considering NIC for miniature bioelectronics one must consider two primary factors: 1) the strength of the magnetic field at the depth of the implant – which depends mainly on the transmitter geometry and current, and 2) how much power can be harvested from a magnetic field of a given strength – which depends on the receiver size and location.

For a circular coil transmitter, the field strength *B* at a distance *z* from the center of the coil can be written as (Equation (1))

(1) $$B_{z}=\frac{\mu_{0}}{2\pi }\;\frac{R^{2}NI}{{\left(z^{2}+R^{2}\right)}^{3/2}}$$

Where *R* is the radius of the coil, *N* is the number of turns in the coil, and *I* is the current through the coil. These three factors can be tailored to optimize for a specific application. Increasing the number of turns or current in a coil will always increase the field strength but changing the radius of the transmitting coil can either increase or decrease the field strength depending on the implant depth. For example, when powered with the same current, smaller coils produce a larger magnetic field at a shallow depth than larger coils. However, because the field strength falls off more slowly as a function of depth from a large coil, large coils are more effective for powering deeper implants. While there are many opportunities to optimize the coil designs to improve performance for a given application, it is generally preferable to increase the radius of the transmitter for deeper implants rather than trying to increase the transmitter current (e.g., Freeman et al.^[^ ^42^ ^]^ (Figure 6C)).

In NIC the magnetic field induces a voltage and current in the receiving bioelectronic that is proportional to the magnetic flux captured by a small, implanted coil. The power generated by an inductively powered implant depends on the area of the pick‐up coil, the number of turns, and the strength of the magnetic field. For a circular coil geometry, the power received at the implant can be written as (Equation (2))^[^ ^16^ ^]^

(2) $$P\;=\frac{\pi^{2}NBR^{3}}{4\mu }\;\times \frac{dB}{dt}$$

Where *N* is the number of turns, *A* is the area of the implanted coil, *R* is the radius of the implanted coil, and *μ* is the permeability of the implanted coil.

Two important design considerations emerge from the received power equation as it relates to miniature bioelectronics.^[^ ^44^ ^]^ First, *A* is the area perpendicular to the magnetic field, so in addition to the physical dimensions of the coil, any angular or translational misalignment will reduce the received power. Second, as a device is made smaller, the area and the number of turns must be reduced. Thus, the received power falls dramatically as a function of radius of the receiver (typically as *R^3^*).^[^ ^16^ ^]^ The received power can also be increased using the transmitter to increase the transverse component of the magnetic field at the implant location (*B_z_*) or the frequency of the applied field (*dB/dt*).

Another important consideration is the resonant frequency, *f*_r_, for which power transfer is maximized. For NIC this resonance is determined by the inductance of the coil (*L*) and the total capacitance of the receiver (*C*), which is typically tuned by changing the value of the capacitor in the circuit (Equation (3)):

(3) $$f_{r}=\frac{1}{2\pi \sqrt{LC}}\;$$

In many cases, inductively powered systems are designed to resonate at 13.56 MHz in the unlicensed industrial, scientific, and medical (ISM) band (other ISM bands, such as 6.78 MHz can also be used). There are three main reasons for this. First, this is a commonly used frequency band and is therefore a practical choice due to the fact that many off‐the‐shelf components are already designed to work in this range. Second, there is a balance between increasing the frequency to increase the power of the implant (Equation (2)) and electromagnetic safety limits which reduce the amount of power that can be safely applied by the transmitter at higher frequencies due to tissue absorption and the range between 1–13 MHz is generally a considered good choice for safe but effective WPT for bioelectronics.^[^ ^25^ ^]^ And finally, even though the power will increase with increasing frequency, some implants also use a ferromagnetic core to increase the captured flux, and these cores become lossy at frequencies higher than this.^[^ ^42^ ^]^ In some cases, these types of implants will even operate at lower frequencies to account for this such as the BION system which operates at 2 MHz and the system used by Maeng et al. which operates at 10 MHz.^[^ ^26^, ^27^ ^]^

So, while in theory a high magnetic field strength at a high frequency applied to a large well‐aligned implanted resonant coil will generate a large amount of power, we see that this is not always safe or practical. When we move away from this idealized picture to more realistic conditions, we can evaluate this type of wireless power transfer in context of the five tradeoffs introduced previously.

Taking these considerations into account we can plot the expected performance envelope for NIC bioelectronics (Figure 5) according to the five performance metrics described in Figure 4. Because the power falls considerably as the devices are miniaturized due to the previously described *R* ^3^ dependence, NIC performs best for relatively larger devices in the range of 5–10 mm in diameter. At these sizes, inductively powered implants can achieve upward of 10 mW of power within safe magnetic field conditions.^[^ ^28^ ^]^ In certain geometries, especially in cases where the coils that power the implant can be wrapped around the implant locations (e.g., peripheral limbs or rodent home cages Figure 6B,D), this technique can achieve good depth and transmitter distance, up to ≈10 cm.^[^ ^26^, ^28^ ^]^ These factors are limited by the maximum field strength the transmitter can safely transmit at typical inductive frequencies and the spatial decrease in field strength. Furthermore, because these implants directly depend on the magnetic flux through the coils, especially for the case of an air core coil, the alignment tolerance reduces to ≈50%. This value is estimated from the fact that magnetic transmitters can be designed to cover large areas of lateral misalignment, but are generally limited in their angular misalignment to angles of less than 45°.^[^ ^29^ ^]^ This means that while NIC implants can operate with some misalignment, they are more suited for applications where alignment is not expected to exceed more than roughly 30–45° for extended periods of time, such as cochlear implants where the two coils are fixed and aligned with each other.

One approach to improve the alignment tolerance is to overpower the implant so that it will still be sufficiently powered even in a lower field strength from misalignment such as in rodent home cage applications.^[^ ^28^ ^]^ For example, Shin et al. demonstrated a NIC system for optogenetics in freely moving mice (Figure 6B). In this case a shallow (0.1 mm) lower power (10 mW) implant in a mouse was powered from up to 10 cm away from loop antennas wrapped around a cage. In another case, Maeng et al. used a more miniature inductive coil (which used a ferromagnetic core) with a length of 2.3 mm implanted 7.8 mm deep in a rat brain. In this case the implant was only able to achieve a maximum power of 0.03 mW and was powered only in one corner of a behavioral area (max transmitter distance of 10 cm).^[^ ^27^, ^42^ ^]^ In order to counteract the poor alignment tolerance of this device in a freely moving rat (50%), they used a combination of three coils and generated a magnetic field in three directions. The BION device is similar to this miniature elongated coil, but due to its larger dimensions and the fact that it is designed to be operated within a coil instead of a distance away, it has slightly improved power (5 mW) and incorporates charge storage components to stimulate at higher power levels.^[^ ^26^ ^]^ This overpowering solution produces an effective solution to improve alignment tolerance at the expense of average power coupling efficiency and may push some applications close to the safety limits for magnetic field intensity. Overall NIC performs best for relatively larger devices that require high power and do not require large angular misalignment tolerance.

### 2.2 Far Field Antenna (RF)

**Figure 7:**
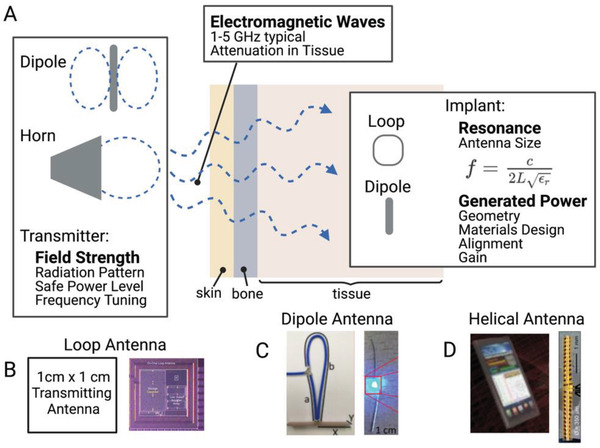
A) Far field wireless power delivery systems use a transmitting antenna to power an implanted antenna using radiating electromagnetic waves. Multiple types of implanted antenna systems have been suggested including B) loop antennas,^[^ ^13^ ^]^ C) dipole antennas,^[^ ^45^ ^]^ and D) helical antennas.^[^ ^46^ ^]^ Adapted with permission.^[^ ^13^ ^]^ Copyright 2017, IEEE; Adapted under the terms of the CC BY license.^[^ ^45^ ^]^ Copyright 2020, the Authors. Published by Elsevier;^[^ ^46^ ^]^ Copyright 2015, Springer Nature.

Another traditional method of wireless power for bioelectronic implants uses far‐field electromagnetic waves from an external antenna to transmit power to a miniature implanted receiving antenna (**Figure** **7**).

While near‐field pickup coils discussed in the previous section can also be classified as loop antennas, there are fundamental differences that separate antennas from near‐field pickup coils as we define them here. A loop antenna operating at low MHz frequencies can almost always be classified as an inductive coil due to the fact that every coil and relevant distance is considerably smaller than the electromagnetic wavelength at these frequencies (≈10–30 m) and thus they operate using the near field magnetic field to power implants. However, as the frequency increases to the GHz range, transmitting antennas (including loop antennas) can be designed with features that are on the scale of a wavelength or fraction of a wavelength. For example, at 2.4 and 5 GHz (common frequency bands) the electromagnetic wavelength is only about 12 and 6 cm respectively. Quarter and half wavelength antennas, though less efficient, are also common. At these higher frequencies, once a device is a few cm away from the transmitter, this is considered the “far‐field” where the electric and magnetic components combine into a radiating electromagnetic (EM) wave. In this regime the power transfer is unlike NIC where the electric and magnetic components were separate and localized. Aside from loop antennas, some other simple types of antennas found in bioelectronic applications include horn antennas and dipole antennas.^[^ ^37^, ^45^, ^46^, ^47^, ^48^, ^49^ ^]^

On the implant side, while loop antennas are primarily powered by the magnetic component of the EM wave, dipole antennas are activated by the electrical component as the standing wave condition is reversed and transformed into electrical power fed to the implant from the center of the antenna. In each case the resonant condition for both the transmitter and receiver is set by the length of the antenna trace. For a half wave dipole antenna this can be written mathematically as:

(4) $$f_{r}=\frac{c}{2L\sqrt{\varepsilon_{r}}}\;$$

Where *L* is the length of the dipole, *c* is the speed of light, and *ε*_r_ is the relative permittivity of the tissue. Aldaoud et al. demonstrated using this type of device to wirelessly light implantable LEDs (Figure 7C).^[^ ^45^ ^]^ Other antenna types such as loop (Figure 7B) and helical (Figure 7D) antennas can also be designed with trace lengths tuned to a specific frequency, making far field antenna one of the most geometrically versatile classes of wireless power.^[^ ^13^, ^46^, ^50^ ^]^

In many cases the types of antenna chosen for the transmitter and receiver depend on multiple factors for each antenna which can include gain, power transfer efficiency, and the radiation pattern. Antenna gain is a commonly reported value and generally refers to a comparison of the measured transmitted or received power of an antenna with an “ideal” antenna. It is measured in units of decibels, dBi or dBd depending on whether the comparison is to an “isotropic” or “dipole” ideal antenna. A higher gain indicates that an antenna can transmit or receive a higher amount of radiated power in a certain direction. Power transfer efficiency is simply the ratio of the power captured by the implant to the power input to the transmitter. Typical values are usually less than 1%.^[^ ^16^ ^]^ These efficiency values are important for antenna power transfer (and also for NIC) because these methods are usually operating near the safety exposure limits, so higher efficiencies are needed to increase the received power at the implant. The radiation pattern is also an important consideration for both the transmitter and receiver as it helps to determine alignment tolerance. Radiation patterns are plots of the gain in the various directions an antenna can radiate and absorb EM waves. For example, as mentioned earlier, horn antennas are highly directional, while dipole antennas are omnidirectional and will therefore have very different radiation patterns (Figure 7). If geometrically feasible, a circularly polarized antenna, which can be formed from two orthogonal dipole antennas, can add greater angular tolerance to these systems.

Implants that use EM power transmission, while not the best choice for transmitting high levels of power, are particularly useful for data transmission to and from an implant. For example, Bluetooth transfer systems use a form of RF data transfer.^[^ ^51^, ^52^, ^53^ ^]^ Systems that use RF power for freely moving rodent experiments also may need to implement a tracking system to efficiently deliver power without exceeding safety limits.^[^ ^50^ ^]^

Propagating EM waves can be advantageous, especially in applications where minimizing losses through distance in air is important. If we look at the practical application space for far field antennas (Figure 5) we first note that the received power is generally low with a maximum around 1 mW due to the low power transfer efficiency. These types of implants can be miniaturized but again with a corresponding drop in efficiency as the fractional wavelength gets smaller. In practical terms, depending on the application, just under one mm is the size devices can shrink to and still receive enough power to power an implant. For example, Rahmani et al. developed a 2.56 mm^2^ loop antenna implant that can be powered by a transmitting loop antenna several cm away capable of generating 1.2 mW of power.^[^ ^13^ ^]^ The depth in tissue an antenna can be implanted is relatively shallow (up to ≈1 cm) due to the safety limits and absorption of EM waves in tissue. Because EM waves at these frequencies can travel long distances in air, the transmitter distance can theoretically be quite large, even if the transferred power is low.^[^ ^54^, ^55^ ^]^ As suggested by the radiation patterns, the alignment tolerance depends on the type of antenna used for transmitting and receiving but is generally low (here we suggest ≈25%, due to the fact that the lateral misalignment and angular misalignment will change the angle of the incident EM wave and reduce the power delivery). This alignment tolerance can potentially be mitigated with tracking algorithms or circularly polarized antennas.^[^ ^13^ ^]^ Overall RF devices are best suited for mm‐cm sized devices implanted at shallow depths below the skin where it is important to achieve reliable long‐range data communication.

### 2.3 Mid Field Inductive Coupling (MDF)

**Figure 8:**
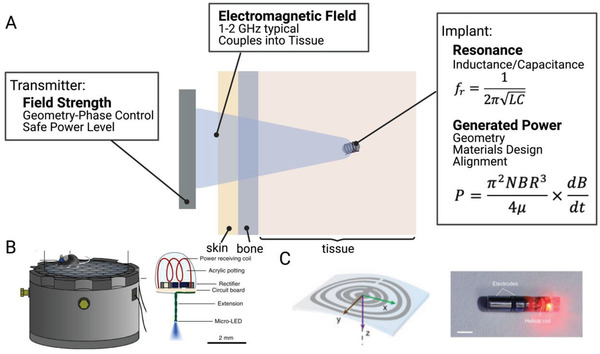
A) Mid‐field wireless power transfer uses an antenna type transmitter to deliver power to an inductively powered stimulator. This method has shown efficacy in B) freely moving mice^[^ ^29^ ^]^ and C) large animal models.^[^ ^15^ ^]^ Adapted with permission.^[^ ^30^ ^]^ Copyright 2015, Springer Nature;^[^ ^15^ ^]^ Copyright 2017, Springer Nature.

A special case of wireless power delivery which combines different aspects of inductive and far‐field methods, mid‐field powering (**Figure** **8**), was demonstrated in mice and pigs by Montgomery et al and Abid et al. and Agrawal et al, respectively.^[^ ^15^, ^30^, ^31^, ^32^, ^33^, ^34^, ^35^, ^36^, ^37^, ^38^, ^39^, ^40^, ^41^, ^42^, ^43^, ^44^, ^45^, ^46^, ^47^, ^48^, ^49^, ^50^, ^51^, ^52^, ^53^, ^54^, ^55^, ^56^, ^57^ ^]^ Traditionally the term “mid‐field” applies to a geometric region in the transition area between near field and far field. However, the methods described here and the physics behind the design deviate somewhat from this standard view.^[^ ^38^, ^58^, ^59^, ^60^ ^]^

In this method a custom designed antenna delivers power to an implanted miniature inductive coil. Though the geometry is smaller, the implant design is fundamentally similar to those discussed in NIC with a small coil of wire attached to a capacitor to determine a resonant frequency.

The transmitters, on the other hand, while relying on some of the principles discussed in RF antennas, are more customized to biological tissue. Rather than solely considering the resonances and wavelengths associated with the antenna hardware design, mid‐field powering focuses on how those properties change in tissue due to the change in the dielectric properties.^[^ ^16^, ^61^ ^]^ Indeed the “mid‐field” nomenclature partially refers to the fact that 1–2 GHz electromagnetic wavelength (*λ*) is shorter in tissue (2–3 cm) than in is in air (15–30 cm), due to the increased dielectric constant (*ε*_r_) in tissue ( $\lambda_{\mathrm{tissue}}=\lambda_{\mathrm{air}}/\left(\sqrt{\varepsilon_{r}}\right)$ (air: *ε*_r_ = 1, tissue: *ε*_r_ ≈50–200).^[^ ^61^ ^]^ This means that if we consider most of the field to be in tissue (as is the case with deeper implants), we are operating at the transition area between near and far field. This allows this method to make use of both the near field and radiating field effects.

More importantly, mid‐field powering makes use of the fact that fields at these frequencies are strongly absorbed by tissue. Normally, this would be avoided for safety concerns, but manipulated properly this means that the tissue can help to transmit, focus, and amplify the original EM waves, allowing for deeper penetration to small implants.

Various mid‐field transmitters have been designed for different applications, however in general, they all follow the same basic principle. A resonant antenna (or cavity) transmits an electromagnetic signal a specific predetermined frequency, which, based on the antenna transmitter design, does not propagate well in air and thus only near field components can be observed. However, when the near field is in close contact with tissue, the field will radiate into the tissue layers as a propagating EM wave due the larger dielectric constant in the tissue.^[^ ^61^ ^]^ This radiating wave can also be focused by further designing the transmitting antenna. The EM wave propagates through tissue and the losses that come not with geometric fall off as in the case of near field, but with losses from absorption of the electric field in tissue. An implant several cm deep in tissue can then be inductively powered by the magnetic component of the EM wave. The power the implant can generate is mostly limited by the amount of incident power that can safely be applied by the transmitter.

In practice these transmitters can range from simple to complex depending on the application. For example, in the case of an experiment in a freely moving mouse, simply tuning the wavelength of the near field applied by a resonant cavity to 1.5 GHz (which is shown to be resonant with a mouse tissue model) was enough to power miniature electrical and optical implants.^[^ ^29^, ^56^ ^]^ In the cases of implants that were deeper in tissue, different tissue layers and field focusing had to be taken into account, which necessitated a more complex transmitter design where phase delays between different ports were used to cause appropriate interference of the original EM waves.^[^ ^15^ ^]^ The optimal EM frequency used also depends on the target application as the dielectric properties can be different for different tissue layers and thicknesses in different areas of the body.^[^ ^49^, ^61^ ^]^

In context of the five tradeoffs, mid‐field wireless power systems show good all‐around performance (Figure 5). Depending on the specific setup, power levels can typically reach a few mW with up to 10 mW for shallower configurations. The size can reduce down to about 1 mm with implantation depths up several cm through skin bone and tissue. The transmitter can be placed a few cm away and does not need to be in direct contact with the tissue. Because this is similar to an inductive effect, the alignment tolerance is similar to an inductive system (50%, see Section 2.1). An added advantage of this technique compared to other recent novel methods at this point in time is its demonstrated versatility in multiple applications across model animals. However, for these different applications one must develop custom transmitters. For example, in freely moving mice (Figure 8B) the depth in tissue was less important, allowing for the other four factors to be more optimized. In a separate case, Agrawal et al. used this method to demonstrate heart pacing in a porcine model. Here, the depth is more important while the transmitter can be placed closer to the skin in a fixed position. Data uplink from these devices has yet to be demonstrated due to the challenges of mixed modality between NIC and RF. The major challenge for human translation is that typically operating powers require transmitted power that is close to the approved safety limits.^[^ ^15^ ^]^ Overall, mid‐field power transfer is a good choice for mm‐sized devices, but because these devices operate near the safety limits using absorbed EM radiation they are typically best for shallow implants or for deep implants that require less than 1 mW average power.

### 2.4 Ultrasound

**Figure 9:**
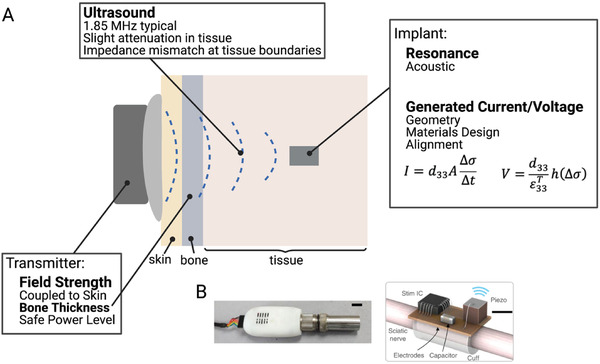
A) Ultrasound wireless power transfer uses an ultrasound transducer in contact with the skin to deliver power to miniature implants, such as those developed by B) Piech et al. Adapted with permission.^[^ ^11^ ^]^ Copyright 2020, Springer Nature.

Recently, ultrasound has emerged as a popular choice for powering mm‐sized bioelectric implants that use piezoelectric receivers to harness acoustic energy.^[^ ^11^, ^62^, ^63^, ^64^, ^65^, ^66^ ^]^ Unlike the previous methods, which used electric and magnetic fields to transfer power, ultrasound uses high frequency sound waves. These waves transmit well through certain types of tissue and are traditionally used for biological imaging but have recently gained popularity as a way to transfer power to miniature implants, such as “neural dust” and “Stim Dust.”^[^ ^11^, ^65^ ^]^ Auricchio et al. also explored ultrasound WPT for human pacemakers (**Figure** **9**).^[^ ^67^ ^]^

While ultrasound excels at delivering power deep in tissue, one drawback is that ultrasound is highly sensitive to impedance mismatches between different types of materials. For example, if the materials have different acoustic properties the ultrasound wave will primarily reflect back instead of being transmitted across the boundary. For bioelectronic applications acoustic reflections often occur at the boundary between air and tissue and, to a lesser extent, boundaries between tissue and bone. Practically, this means the ultrasound transmitter must be placed in direct contact with the skin, acoustically coupled by placing a gel between the transmitter and skin. It also cannot operate in the areas of the body around the lungs.^[^ ^68^, ^69^ ^]^ In areas completely encased in bone, such as the brain, transmission can be complicated and will require specially designed focused ultrasound transmitters.^[^ ^70^, ^71^ ^]^ In more simple peripheral nerve applications researchers can usually use traditional readily available ultrasound transmitters.

Ultrasound powered implants usually contain a piezoelectric element which transduces the ultrasound energy to electrical energy through a vibrating resonant mode. The piezoelectric materials develop a voltage across them in response to the applied stress by the incident ultrasound waves. For example, unstressed lead zirconate titanate (PZT), a commonly used piezoelectric material, has a cubic crystal form with Ti or Zr at a neutral point in the center of the cube. However, under mechanical force, the crystal shape changes and displaces the central Ti or Zr and the crystal develops an electrical polarization and generates electrical energy.^[^ ^72^ ^]^ In general, the voltage a piezoelectric material generates can be written as (Equation (5)):^[^ ^73^ ^]^

(5) $$V\;=\frac{d_{33}}{\varepsilon_{33}^{T}}\;h\left(\Delta \sigma \right)$$

From this expression the relevant factors to determine the voltage a piezoelectric material will generate are the thickness *h*, the applied stress Δ*σ*, and two material constants *d*_33_ and $\varepsilon_{33}^{T}$. *d*_33_ is a piezoelectric coefficient and $\varepsilon_{33}^{T}$ is the permittivity (which is related to the capacitance of the material). The subscripts for these two constants depend on the directional relationship between the polarization and the applied stress. The most common modes are *d*_31_/*ε*_31_ (for polarization across the thickness and stress applied along the length) and *d*_33_/*ε*_33_ (for polarization and stress applied across the thickness). Other modes are also possible, but less likely to be used in biological applications. One thing to note about the voltage is that the only geometric dependence is on the thickness of the piezoelectric material, which gives an advantage for miniature devices compared to an implanted coil where the voltage depended on the area.

An expression for the current from a piezoelectric can also be written (Equation (6)):^[^ ^73^ ^]^

(6) $$I\;=d_{33}\;A\frac{\Delta \sigma }{\Delta t}$$

As expected for a capacitive material, here we see a dependence on the area (*A*) of the material. Another thing to note for implant design is that both of these expressions highly depend on the piezoelectric coefficient. Indeed, the reason PZT is so commonly used is due to its coefficient of 100–500 pC N^−1^, which is relatively large compared to other materials.^[^ ^74^ ^]^

Similar to other types of implants, the available power an implant can generate can be increased under resonant conditions, which exist for the different modes described earlier. The resonances used in piezoelectric implants are similar in fundamentals to those used in far field antennas, where the size of the device is on the order of a wavelength. However, in this case we must consider the acoustic wavelength which is 10^5^ times smaller than the EM wavelength for a given frequency where the acoustic speed in tissue is ≈1540 m s^−1^.^[^ ^65^ ^]^ This means that resonant piezoelectric devices can be made much smaller and more efficient than an EM antenna at the same frequency. The safety standards are also different (and higher) for ultrasound waves since they are not absorbed by the body as easily as EM waves.^[^ ^25^, ^75^ ^]^

Any bioelectronic implementation using ultrasound as the wireless power method can potentially suffer from the tradeoff of having to put the transmitter in contact with the skin (zero transmitter distance) (Figure 5). However, it has advantages in miniaturization over more traditional methods due to the acoustic resonance with the potential for future sub‐mm size devices. The other tradeoffs of power (several mW), depth (up to ≈1–2 cm), and alignment tolerance (≈50%) are moderate and within the bounds of most bioelectronic applications. For example, a “Stim Dust” implant was used to stimulate the sciatic nerve of a rodent model in a proof‐of‐principle demonstration.^[^ ^11^ ^]^ Because the transmitter is already approved for imaging applications, ultrasound may be advantageous for use in peripheral targets in humans where bone and air boundaries are less of an issue. Because the coupling to ultrasound‐powered devices is very sensitive to alignment errors of a few millimeters and the transmitters require ultrasound gels or foams, these devices may be best for applications that do not need continuous power like diagnostics and monitoring. Overall, ultrasound is a good choice for mm‐sized devices implanted several centimeters below the skin where there is no need for long‐term continuous power or powering through bone.

### 2.5 Magnetoelectric

**Figure 10:**
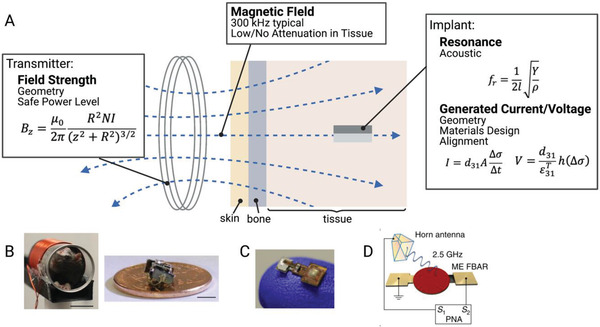
A) Magnetoelectric wireless power transfer transfers power from an external coil to an implanted magnetoelectric device. This system has been demonstrated in B) freely moving rats and can be used with C) integrated circuits.^[^ ^23^, ^39^ ^]^ D) Miniature magnetoelectric devices can also be activated using an EM antenna.^[^ ^37^ ^]^ Adapted with permission.^[^ ^23^ ^]^ Copyright 2020, Elsevier;^[^ ^16^ ^]^ Copyright 2020, IEEE. Adapted under the terms of the CC BY license.^[^ ^37^ ^]^ Copyright 2017, the Authors. Published by Springer Nature.

Another type of “hybrid” wireless power recently demonstrated uses the magnetoelectric effect to transfer power to small bioelectronic implants. This method combines some of the miniaturization advantages of ultrasound with the tissue penetration advantages of magnetic fields. Specifically using a magnetic field delivered by a near field coil or a far field antenna it is possible to activate an implanted magnetoelectric material, usually in the form of a thin bilayer film that converts an alternating magnetic field into an alternating electric field (**Figure** **10**). Typically, this conversion is made by a bilayer film where one layer contains a magnetostrictive material (which generates stress in an alternating magnetic field) and the other layer is a piezoelectric material that converts stress into a voltage. Thus, in the presence of a magnetic field, this device will generate stress in the magnetostrictive layer which transfers to the piezoelectric layer and the resulting voltage can power to an implant similar to an ultrasonically powered piezoelectric crystal described in the previous section.

Like ultrasound‐powered devices, ME devices also operate under resonant conditions for the piezoelectric material, meaning that they operate at the acoustic resonant frequency of the energy harvesting material. While this fact enables scaling to millimeter and sub‐millimeter sizes, it also means that these devices often do not operate at the frequencies of commercially available transmitters. As a result, ME WPT systems typically require the design of custom transmitters. In the works demonstrated by Singer et al. and Yu et al., the resonant frequency was primarily dependent on the length of the implanted film (Equation (7))^[^ ^76^ ^]^

(7) $$f_{r}=\frac{1}{2l}\;\sqrt{\frac{Y}{\rho }}$$

Here we see the length, *l*, dependence along with a number of material properties (*Y*, Youngs modulus, *ρ*, density). This acoustic resonant condition can be additionally advantageous in comparison with other magnetically powered implants because magnetic fields can be easier to generate and can operate at a much higher safety limit at these lower frequencies.^[^ ^16^ ^]^

In addition to the transmitter design (discussed in NIC and RF) and the choice of piezoelectric design (discussed in ultrasound), magnetoelectrics have the added design consideration of the magnetostrictive material, which is characterized by the magnetostrictive coefficient *λ*. *λ* is an experimentally measured parameter which compares the fractional change in length with an applied magnetization. The derivative of this curve gives the piezomagnetic coefficient *d_ij_* which is the magnetic analog of the previously discussed piezoelectric coefficient.

Maximizing this piezomagnetic coefficient is a combination of two factors. The first is choosing a material with a large change in *λ*. This may not always be equivalent to choosing a material with a large magnetostriction, because it is the derivative that determines the piezomagnetic coefficient. Secondly, in many cases a bias magnetic field is required because the derivative will be maximized at the inflection point of the magnetostriction curve.^[^ ^77^ ^]^ Adding in a bias field allows an alternating field to oscillate around that inflection point and generate the maximum possible strain to be transferred to the piezoelectric. The strength of the bias field depends on the material used. Metglas and Terfenol‐D, with piezomagnetic coefficients of 1000 and 57 nm A^−1^ respectively, are two commonly used magnetostrictive materials in magnetoelectric films.^[^ ^77^, ^78^, ^79^, ^80^, ^81^ ^]^

While the main in vivo demonstrations of magnetoelectric wireless power transfer for bioelectronic applications use lower frequency magnetic fields (100s of kHz, Figure 10B,C), Nan et al., has shown further miniaturization of ME films to miniature antennas which can be activated by a high frequency RF transmitting antenna (Figure 10D).^[^ ^23^, ^38^, ^39^ ^]^ These two different techniques are similar, yet because the transmitter is different, they have slightly different tradeoffs and application spaces.

For the antenna transmitter version of an ME power system, the main advantage lies in the miniaturization, with feature sizes on the order of hundreds of microns in the GHz range (Figure 5). However, this miniaturization comes with lower power levels compared to other larger acoustic techniques. The depth is again limited by safe levels of RF exposure and because the power levels are already low, the alignment tolerance will likely be reduced compared to systems with higher power levels. Magnetoelectric systems powered by a lower frequency magnetic coil can achieve higher power levels (several mW) with a larger device footprint (feature sizes on the order of several mm). Because magnetic fields at these frequencies can travel attenuated through air and tissue the depth and transmitter distance can easily approach several cm. These ME devices also have slightly improved alignment tolerance due to the fact that the magnetostrictive layer helps to capture and concentrate the magnetic field along the length of the implant.^[^ ^16^, ^78^ ^]^ However, this magnetostrictive layer may also cause issues with MRI compatibility in future devices. In addition, data uplink has yet to be demonstrated, and it may be challenging to develop a passive backscatter mechanism like what has been shown for NIC, RF, and US uplinks. Overall, magnetoelectrics is an excellent choice to power devices that are mm‐sized or smaller particularly if the application requires high‐tolerance to translational misalignments or power transfer through air and bone.

### 2.6 Light

**Figure 11:**
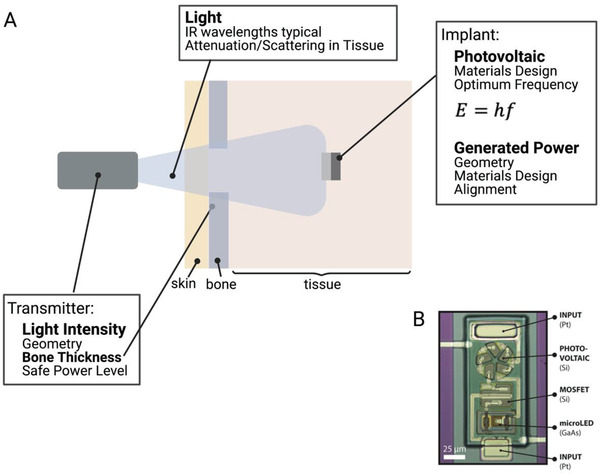
A) Wireless power transfer to implanted photovoltaics using light. B) While transmission through bone can be a challenge, this technique can be used to power miniature implants.^[^ ^12^ ^]^

Light is another method to deliver wireless power, and although it uses EM waves, the receiver physics and extremely short wavelengths gives it unique advantages compared to RF power. In particular, power harvesting by the receiver is typically achieved not by an antenna, but by a photovoltaic component that absorbs light within a specific wavelength range (**Figure** **11**).

Light can be in the form of sunlight (which contains many frequencies of light) or from a source with a narrow frequency band such as a laser or LED.^[^ ^12^, ^53^, ^82^, ^83^ ^]^ The photovoltaic receiver is most commonly made from silicon, which means that the ideal frequency of light is about 2.7 × 10^5^ GHz (1100 nm) which is in the near infrared range. This is determined based on the fact that the incident photons must have energy to knock electrons free within the material. This minimum amount of energy is the band gap energy and in silicon it is 1.78 × 10^−19^ J. The energy of a photon, which determines the optimum frequency is calculated using Planck's law (Equation 8):

(8) E=hf

Where *E* is the energy, *h* is Planck's constant, and *f* is the frequency of the light. In theory any frequency with energy greater than the band gap energy will free electrons (i.e., higher frequencies/shorter wavelengths), but in practice if the energy is too high, the electrons are knocked completely free of the material and are no longer useful. These near infrared frequencies are advantageous to bioelectronic applications because they can penetrate through tissue better than other frequencies of visible light. In fact, the “near infrared window” (wavelengths of ≈700–2500 nm) is an optimal range for tissue penetration due to minimal absorption in tissue at these wavelengths. Within this range photon penetration is primarily a function of scattering allowing for deeper penetration than wavelengths limited by absorption in blood and water outside of this window.^[^ ^84^ ^]^

These free electrons in the silicon are used to form a voltage across the thickness of the silicon which can drive implanted circuits. For this to happen the silicon must be doped with another material so charge can build up. There are two types of doping n‐type and p‐type. N‐type doping gives the silicon an excess of negatively charged electrons and p‐type gives an excess of positively charged holes (or lack of electrons). When these two materials are placed in contact with each other a p‐n junction is created with a “depletion zone” in the p‐type material near the boundary. In this area the excess electrons from the n‐type material pair with the holes in the p‐type material. This electron‐hole pair is what is knocked free by incoming photons. When this happens the electron and hole separate and travel to opposite sides of the material where the electrons can flow through the wires attached between the top and bottom of the silicon.

A major advantage of photovoltaic power generation is that because the wavelength of light is on the order of 1 µm, these types of devices can be miniaturized to sub mm areas. The drawback of this extreme miniaturization is the fact that the power scales linearly with the area of the depletion region (Figure 5). Because the implant power is generally low, any significant drop in efficiency, estimated to happen at angles >45° (AT ≈50%), will likely lead to losses in device functionality.^[^ ^85^ ^]^ The depth at which one can use light for power delivery is primarily limited by the scattering and absorption by the biological environment, which can vary depending on the thickness, type of tissue and presence of bone. In theory, because these devices could be powered by a laser, the transmitter distance can theoretically be tens of centimeters, however the distance below the skin is typically limited to a few mm due to optical scattering. For example, Cortese et al., used this wireless power technology to power miniature temperature sensors in a mouse brain (Figure 11B).^[^ ^12^ ^]^ Overall, light is often the best choice for low‐power, sub‐mm‐sized implants at depths of several mm below the skin.

### 2.7 Other Techniques

In addition to the major WPT technologies that have been reduced to practice, there are several emerging methods that have been proposed but have yet to be demonstrated and well‐characterized in vivo.

*Capacitive Coupling*: Capacitive coupling relies on two planar electrodes on an implant and two planar electrodes outside the body in either a monopolar or bipolar configuration.^[^ ^41^, ^86^ ^]^ When a current is applied to the electrodes outside the body it acts as one side of a parallel plate capacitor, with the second side being the implanted electrode. Thus, the power travels wirelessly through the body between the two plates. The other internal and external plates are required to complete the circuit. This method generally requires a short distance between the transmitter and receiver and can require large electric fields between the plates which can interact with tissue and lead to hazardous exposure. Thus, capacitive coupling appears best positioned for low power applications, especially when angular misalignment would prevent the use of other techniques.

*Self‐Powered Devices*: Various other techniques have been proposed that differ from the traditional wireless transmitter‐receiver design by converting some form of energy naturally generated in the body to electrical energy instead of using an external transmitter. One of the simplest forms of this type of device are piezo mechanical devices.^[^ ^87^, ^88^, ^89^ ^]^ In this case an implanted piezoelectric generates energy when the body moves rather than relying on incoming ultrasound or magnetic fields to activate it. Other types of devices use electrical or chemical gradients in the body to generate power.^[^ ^52^, ^90^, ^91^ ^]^

These types of devices can be less consistent because there is no way to externally control the amount of power they will generate, and it will be difficult to operate under any sort of resonant condition. The power will therefore be fixed based on the biological environment. This may make these types of devices unsuitable for some applications such as heart pacing, where the timing is crucial. However, as implant circuitry and charge storage methods become more developed, these types of implants will be able to expand their application space.

## 3 Size and Safety Considerations

While it is challenging to make direct unbiased comparisons across such a diverse array of wireless power technologies, we can directly compare the physical size of the devices, the amount of power generated, and, when reported, how close these systems are to the safety limits. These safety limits are generally set to prevent a rise in tissue temperature above ≈1 C. This sets a limit on how much energy the tissue can absorb, which is quantified as the specific absorption rate (SAR) limit. However, in some cases the SAR level cannot be measured, and the transmitter is operating from a distance away, which can occur in RF and NIC systems. In that case, an electric field limit can be used instead of SAR to ensure safety. This electric field limit is also important in cases where the implant receives a strong electric field at frequencies <5 MHz, as found in some NIC and ME systems. In this case, the electric field drives heating of the tissue, and one should refer to the maximum electric field limit as well as the SAR limit.^[^ ^25^ ^]^

**Figure 12:**
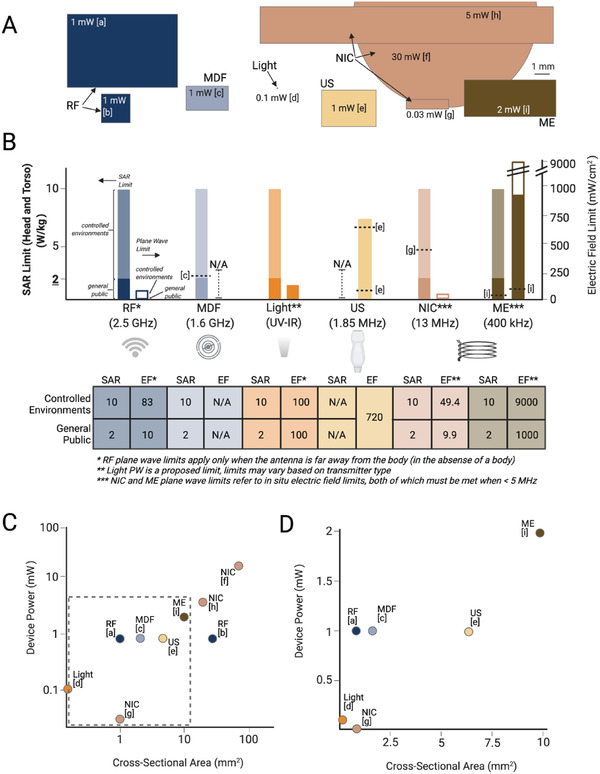
A) Comparisons of the size, power, and safety limits of miniature devices used in in vivo experiments shows a range of devices sizes and powers. B) The safety limits vary for each type of WPT and, in some cases, devices already operate at or near the limits which limits the transmitter power. C,D) Increasing the size of the implant can also increase the generated power. (References: a^[^ ^50^ ^]^ b^[^ ^13^ ^]^ c^[^ ^15^ ^]^ d^[^ ^12^ ^]^ e^[^ ^11^ ^]^ f^[^ ^14^ ^]^ g^[^ ^42^ ^]^ h^[^ ^26^ ^]^ i^[^ ^23^ ^]^)

For each transmitter type there are typically two standard safety limits: one for the general public, and one for controlled environments like a medical clinic. The electric field safety limits are set based on the type of transmitter and the frequency. **Figure** **12** shows the general public and controlled environment limits for each type of transmitter discussed above.^[^ ^25^, ^75^, ^92^, ^93^ ^]^ The SAR limits are the same for all types of transmitters 2 W kg^−1^ for the general public and 10 W kg^−1^ for controlled environments in head and torso applications. These limits are higher in the limbs. When available, we also noted the levels used by reported devices in in vivo experiments.^[^ ^11^, ^15^, ^16^, ^23^, ^42^ ^]^ In some cases, the devices may also be functional at lower exposure levels and a higher level was used, possibly to ensure in vivo functionality.

There are several conclusions we can draw from these safety limits. The electric field limits are significantly lower for electromagnetic and higher frequency magnetic forms of power transfer. Furthermore, these low levels combined with the fact that existing technologies in these categories already exceed the general public SAR limits, means that, at least in clinical applications, these types of technologies as described will be limited to controlled environment use like in treatment clinics or preclinical animal use only. We also note that in some cases, implants may be able to operate in a pulsed mode where the instantaneous power may be over a safety limit, but the average power is within the specified limits. Furthermore, while ultrasound like the other methods can cause heating if it exceeds FDA limits, one must also consider mechanical effects such as cavitation.

Figure 12A also shows a to‐scale comparison of the sizes of various devices described above and the approximate maximum power each device could generate in vivo.^[^ ^11^, ^12^, ^13^, ^15^, ^23^, ^26^, ^29^, ^42^, ^50^ ^]^ Though it is difficult to draw too many quantitative conclusions, in general we can see that the larger devices generate more power (Figure 12C,D). Furthermore, we can see that many methods of wireless power transfer can currently activate implants that are in the 1–10 mm^2^ size range. Future progress in many of these techniques will push these miniaturization limits to reach new biological targets.

## 4 Conclusions and Future Directions

**Figure 13:**
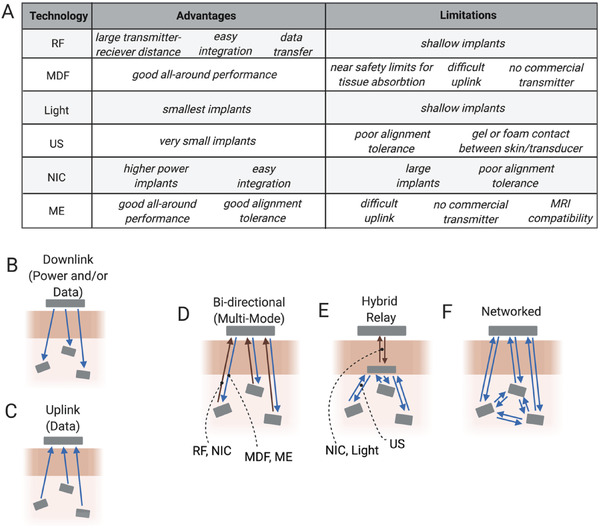
A) Summary of the main advantages and limitations of each WPT method can guide future applications for each modality. Current devices generally use only up to several channels or devices to either B) stimulate or C) record. D–F) Future iterations of bioelectronic devices may combine different modalities to increase channel count and device applications.

Although there are many metrics one must consider when choosing the right wireless power option for a particular implanted bioelectronic device we attempted here to provide a guide for making this choice and a discussion of the tradeoffs among the six major types of wireless power technologies that have been demonstrated in vivo. **Figure** **13**A summarizes the main advantages and limitations of each of the WPT types discussed here.

Going forward one can consider what types of systems might be possible by using these types of wireless power technologies alone or in combination (Figure 13B–F). Thus far most wireless power transfer techniques have focused on transmission between a single transmitter and a single implanted device in one or more animals to either stimulate or record (Figure 13B,C). In most cases each implant has one stimulation or recording channel, with a few that have two to three channel interfaces for each device. However, many wired or battery powered devices used today use a minimum of four channels for stimulation, and sometimes many more.^[^ ^94^, ^95^, ^96^ ^]^ To achieve these higher channel counts it may be necessary to create networks of multiple wireless bioelectronic implants. These networks may one day mix methods for wireless power and data transfer and could form any number of network architectures (Figure 13D–F).^[^ ^40^, ^97^, ^98^, ^99^ ^]^ These multimode systems can combine the advantages and minimize the limitations of the various WPT techniques. Free from wire and tethers these networks could span large areas of tissue and coordinate multimodal sensory and simulation capabilities to provide precise regulation of physiological processes.^[^ ^100^ ^]^ Indeed, bioelectronics empowered by a suite of wireless data and power transfer technologies is certain to usher in innovative minimally invasive and distributed systems for improving the way we understand and treat disease.

## Conflict of Interest

The authors declare no conflict of interest.
